# The effects of a lifestyle-focused text-messaging intervention on adherence to dietary guideline recommendations in patients with coronary heart disease: an analysis of the TEXT ME study

**DOI:** 10.1186/s12966-018-0677-1

**Published:** 2018-05-23

**Authors:** Karla Santo, Karice Hyun, Laura de Keizer, Aravinda Thiagalingam, Graham S. Hillis, John Chalmers, Julie Redfern, Clara K. Chow

**Affiliations:** 1 The George Institute for Global Health, Sydney, Australia; 2 Faculty of Medicine and Health, The University of Sydney, Sydney, Australia; 3 Westmead Applied Research Centre, Westmead Clinical School, The University of Sydney at Westmead Hospital, PO Box 154, Westmead, NSW 2154 Australia; 4 Cardiology Department, Westmead Hospital, Sydney, Australia; 5 Westmead Institute for Medical Research, Sydney, Australia; 6 School of Medicine and Pharmacology, University of Western Australia, Perth, Australia; 7 Department of Cardiology, Royal Perth Hospital, Perth, Australia

**Keywords:** Diet, Mobile phone, Text-messages, Text-messaging, mHealth, eHealth, Randomised controlled trial

## Abstract

**Background:**

A healthy diet is an important component of secondary prevention of coronary heart disease (CHD). The TEXT ME study was a randomised clinical trial of people with CHD that were randomised into standard care or a text-message programme in addition to standard care. This analysis aimed to: 1) assess the effects of the intervention onadherence to the dietary guideline recommendations; 2) assess the consistency of effect across sub-groups; and 3) assess whether adherence to the dietary guideline recommendations mediated the improvements in objective clinical outcomes.

**Methods:**

Dietary data were collected using a self-report questionnaire to evaluate adherence to eight dietary guideline recommendations in Australia, including consumption of vegetables, fruits, fish, type of fat used for cooking and in spreads, takeaway food, salt and standard alcohol drinks. The primary outcome of this analysis was the proportion of patients adhering to ≥ 4 dietary guideline recommendations concomitantly and each recommendation was assessed individually as secondary outcomes. Data were analysed using log-binomial regression for categorical variables and analysis of covariance for continuous variables.

**Results:**

Among 710 patients, 54% were adhering to ≥ 4 dietary guideline recommendations (intervention 53% vs control 56%, *p* = 0.376) at baseline. At six months, the intervention group had a significantly higher proportion of patients adhering to ≥ 4 recommendations (314, 93%) compared to the control group (264, 75%, RR 1.23, 95% CI 1.15–1.31, *p* < 0.001). In addition, the intervention patients reported consuming higher amounts of vegetables, fruits, and fish per week; less takeaway foods per week; and greater salt intake control. The intervention had a similar effect in all sub-groups tested. There were significant mediational effects of the increase in adherence to the recommendations for the association between the intervention and LDL-cholesterol (*p* < 0.001) and body mass index (BMI) at six months follow-up (*p* = 0.005).

**Conclusion:**

A lifestyle-focused text-message programme improved adherence to the dietary guideline recommendations, and specifically improved self-reported consumption of vegetables, fruits, fish, takeaway foods and salt intake. Importantly, these improvements partially mediated improvements in LDL-cholesterol and BMI. This simple and scalable text-messaging intervention could be used as a strategy to improve diet in people with CHD.

**Trial registration:**

Australia and New Zealand Clinical Trials Registry ACTRN12611000161921. Registered on 10 February 2011.

**Electronic supplementary material:**

The online version of this article (10.1186/s12966-018-0677-1) contains supplementary material, which is available to authorized users.

## Background

Cardiovascular disease (CVD), including coronary heart disease (CHD), is the leading cause of death worldwide. [1, 2] Both CHD and other CVDs can be prevented through adherence to medical therapy and a healthy lifestyle, which includes absence of tobacco use, regular physical activity and a healthy diet. The role that a healthy diet plays in primary and secondary prevention of CVD has been studied and well-established in the last two decades. [3–6] Recently, food-based dietary patterns have been emphasised in dietary guidelines in many countries, replacing the previous emphasis on nutrients. [7–10] The Mediterranean diet and the DASH (Dietary Approaches to Stop Hypertension) diet are two types of dietary patterns that have been shown to prevent major cardiovascular events and sudden cardiac death, and to reduce cardiovascular risk by lowering blood pressure (BP), respectively. [11–19] These diets share common factors of promoting intake of fruits and vegetables, whole grains, nuts, fish and, vegetable and olive oils. [12, 20]

In our globalised world, however, traditional dietary patterns in many developed and developing countries have been shifting to a Western diet rich in animal products and refined carbohydrates and low in whole grains, fruits and vegetables in the last two decades. [21] To reverse this shift, it is important to promote the consumption of some food items that have been linked with CVD prevention. Increasing consumption of vegetables and fruits is essential, as these food items have been associated with lower CHD risk and CVD mortality. [20, 22] An increase in vegetable and fruit consumption up to 600 g per day has been estimated to reduce the burden of CHD by 31%. [23] In addition, fish consumption has been shown to reduce CHD mortality in high-risk populations. [24–26] In contrast, a diet high in salt and saturated fats is associated with increased CVD risk. [5, 27] Therefore, current national and international dietary guidelines recommend a diet high in vegetables, fruits and fish, and low in salt, as well as replacing saturated with unsaturated fats. [7–10] To ensure adherence to these guidelines, interventions to recommend and reinforce healthy eating habits are essential, if we are to reduce CVD recurrence as well as improve morbidity and mortality from other health conditions globally.

Text-messaging can be a quick low-cost way of promoting CVD prevention by motivating and reinforcing a healthy eating habit. The Tobacco, Exercise and Diet Messages (TEXT ME) trial was a randomised clinical trial (RCT) of a lifestyle-focused text-messaging support programme delivered for six months to patients with CHD. The TEXT ME main results are reported elsewhere. [28] In summary, compared to the control group at six months, the intervention group achieved significantly lower levels of LDL-cholesterol, systolic BP, body mass index (BMI), smoking rates and higher physical activity levels. In addition to the above-mentioned outcomes, dietary data were prospectively collected and diet was pre-specified as a secondary outcome of the TEXT ME study. [29] This paper presents the analyses of these data; the aims were to analyse the dietary data to: 1) assess the effects of the TEXT ME intervention on adherence to the dietary guideline recommendations, both combined and individually; 2) assess the consistency of effect of the TEXT ME intervention across sub-groups; and 3) assess whether adherence to the dietary guideline recommendations mediated the improvements in objective clinical outcomes.

## Methods

### Study design

The TEXT ME study was a parallel-group single-blind, RCT of 710 patients with proven CHD that were randomised into standard care or a text-message intervention in addition to standard care. [29] This paper presents an analysis of the TEXT ME study using the dietary data collected in study. In this analysis, eight food items were assessed based on the recommendations from the National Health and Medical Research Council (NHMRC) Australian Dietary Guidelines [9] and the National Heart Foundation of Australia (NHFA) Heart-Healthy Eating Tips . [30] The study was approved by the Western Sydney Local Health District Human Research Ethics Committee and all patients provided written informed consent. The trial was registered in the Australia and New Zealand Clinical Trials Registry (ACTRN12611000161921) on 10 February 2011.

### TEXT ME study intervention

The text-message development process and the study intervention were previously detailed and are briefly described here. [29, 31] The intervention group received four text-messages per week, including at least one message per week focussing on diet, for six months in addition to standard care. The text-messages were semi-personalised and provided advice, motivational reminders and support to change lifestyle behaviours. The messages’ content was based on the Australian Heart Foundation secondary prevention guide [32] and developed in four modules comprising key secondary prevention areas: general cardiovascular health, smoking, physical activity and diet. The text-messages in the diet module aimed to provide general healthy eating tips and motivate patients to eat more fruits and vegetables, increase fish intake, decrease unhealthy fat use and decrease the levels of salt consumption in their diet (Table 1).

**Table 1:** Examples of diet text-messages used in the TEXT ME Study

*General healthy eating tips*	
Hello <xxx>, reduce your plate size to help limit portion size.	
*Vegetables and fruits intake*	
Hi < xxx>, healthy eating means at least 5 serves of vegetables & 2 serves of fruit every day.	
*Fish intake*	
<xxx>, fish & seafood contain omega-3 fats which help reduce the risk of heart disease & stroke.	
*Unhealthy fat use*	
Try steaming, baking, or BBQ to reduce the need for excess oil when cooking.	
*Salt intake*	
Try avoiding adding salt to your foods by using other spices and herbs.	

Note: <xxx> represents the patient preferred name

BBQ, Barbecue

### Assessment of dietary data

Dietary data were collected at baseline and at six months using a 10-item self-report questionnaire developed for this study based on the World Health Organisation (WHO) STEPS (STEPwise approach to chronic disease risk factor Surveillance) instrument. [33] The questionnaire was designed to estimate the patient’s consumption of fruit, vegetables, fish, oil and fat, and control of salt intake. Using these questions, we aimed to assess how well the patients were adhering to the recommendations of the NHMRC Australian Dietary Guidelines [9] and the NHFA Heart-Healthy Eating Tips. [30] The key dietary guidelines’ recommendations assessed in this study are presented in Table 2.

**Table 2:** Key dietary recommendations in Australia

Food item	NHMRC Australian Dietary Guidelines Recommendations [9]	NHFA Heart-Healthy Eating Tips Recommendations [30]
Vegetables	Eat at least 5 serves^a^ of vegetables every day	Eat 5 serves^a^ of vegetables per day
Fruits	Eat at least 2 serves^b^ of fruit every day	Eat 2 serves^b^ of fruit per day
Oils	Replace saturated fat oils (coconut and palm oils) with unsaturated fat oils, including polyunsaturated (sunflower, soybean, sesame, corn and grape seeds oils) and monounsaturated fats (canola, nut, rice bran or olive oil)	Use canola, sunflower, soybean or olive oil when cooking
Spreads	Use small amounts of unsaturated spreads and oils instead of butter	Use margarine or oils instead of butter
Fish	Eat around 2 serves^c^ of fish per week	Eat fresh or canned fish two to three times a week (salmon, tuna, sardines)
Takeaway foods	Limit takeaway to once a week or less	Eat less takeaway food
Salt	Limit intake of foods and drinks containing added salt	Choose ‘reduced salt’ or ‘no added salt’ foods
Alcohol drinks	Drink no more than 2 standard drinks containing alcohol on any one day	x

*NHFA* National Heart Foundation of Australia, *NHMRC* National Health and Medical Research Council

^a^One serve of vegetables is ½ cup of cooked vegetables or 1 cup of salad

^b^One serve of fruit is 1 medium piece or 2 small pieces of fresh fruit, or one cup of chopped or canned fruit

^c^One serve of fish is 100 g of cooked fish fillet (about 115 g raw) or one small can of fish

Dietary data on consumption of vegetables, fruits, type of oil for cooking, takeaway meals and salt intake control were collected using the TEXT ME diet questionnaire (Additional file 1), in the same manner that they are collected using the WHO STEPS instrument (Additional file 2). In addition, the TEXT ME diet questionnaire had questions about fish consumption and the type of oil or spread used on bread. In the assessment of type of oil for cooking, vegetable and olive oils were considered as poly and monounsaturated fat oils, as well as other answers that specified the use of canola, sunflower, soybean, corn, grapeseed, rice bran, or nuts oils. The patients were also asked whether they consumed alcohol once a week or more for most weeks of the year and if so, how many standard drinks they would have in a typical week, including wine, beer and spirits.

### Adherence to dietary guideline recommendations

Self-reported adherence to eight items of the dietary guideline recommendations was assessed using the key recommendations from the NHMRC Australian Dietary Guidelines and the NHFA Heart-Healthy Eating Tips as shown in Table 2. Adherence to each dietary guideline recommendation was computed as follows: 1) Consumption of ≥ 35 serves of vegetables per week (≥ 5 serves per day for 7 days a week); 2) Consumption of ≥ 14 serves of fruits per week (≥ 2 serves per day for 7 days a week); 3) Use of poly and monounsaturated fat when cooking, including canola, sunflower, soybean, corn, grapeseed, rice bran, olive or nuts oils; 4) Use of margarine and unsaturated fat on bread; 5) Consumption of ≥ 300 g of fish per week (300 g ~ 2 serves per week); 6) Consumption of takeaway food once a week or less; 7) Salt intake control on a regular basis; and 8) Consumption of ≤ 14 standard alcohol drinks per week (≤ 2 standard drinks per day for 7 days a week). No partial adherence to the dietary guideline recommendations was computed. The primary outcome of this analysis was pre-specified as the proportion of patients adhering to ≥ 4 recommended dietary guideline items evaluated in this study. In addition, the mean number of dietary guideline recommendations achieved was assessed.

Each dietary guideline recommendation item was also assessed individually as secondary outcomes of this analysis. Each recommendation was analysed as the proportions of patients adhering to the individual recommendation using the same criteria presented above. However, to provide more information about the different levels of vegetables and fruits consumption, four levels of serves of vegetables and fruits were analysed as serves consumed on: 1) 7 or more days per week (≥ 35 serves/week for vegetables and ≥ 14 serves/week for fruits); 2) 5 to < 7 days per week (25–34 serves/week for vegetables and 10–13 serves/week for fruits); 3) 3 to < 5 days per week (15–24 serves/week for vegetables and 6–9 serves/week for fruits); and 4) less than 3 days per week (< 15 serves/week for vegetables and < 6 serves/week for fruits). In addition, mean serves of vegetables, mean serves of fruit, mean grams of fish, mean number of takeaways meals and mean number of drinks consumed per week were assessed.

### Analysis

All analyses were performed using SAS statistical software version 9.4 (SAS Institute Inc). Summaries of baseline continuous variables were presented as means and standard deviations and categorical variables were presented as frequencies and percentages. To analyse the effect of the TEXT ME intervention on adherence to dietary guideline recommendations at six months, the proportion of patients adhering to ≥ 4 recommendations was analysed in terms of relative risk (RR) at six months and compared between groups using a log-binomial regression. In addition, each dietary guideline recommendation was analysed individually. Continuous variables were analysed in terms of mean difference using analysis of covariance (ANCOVA), while categorical variables were analysed in terms of RR using log-binomial regression, where the baseline values of the analysed parameters were used as covariates where appropriate. Missing data was not replaced or imputed.

To examine the consistency of effect of the TEXT ME intervention on the primary outcome across the subgroups, we performed a log-binomial regression model including an interaction term between intervention, subgroups and the main effects to assess whether there was significant heterogeneity of effect across sub-groups, including age, gender, education level, BMI, smoking status and cardiac rehabilitation attendance. No adjustments were made for the sub-group analyses. The choice of sub-groups was guided by discussions with the clinical investigators, in which there was a consensus that it was important to evaluate heterogeneity of effects between older vs younger (> 60 years vs ≤ 60 years), male vs female, higher vs lower education (> 13 years vs ≤ 13 years), higher vs lower BMI (≥ 25 kg/m^2^ vs < 25 kg/m^2^), smokers vs non-smokers and cardiac rehabilitation attenders vs non-attenders, as some of these characteristics have been related to poorer diet. The adherence to ≥ 4 dietary guideline recommendations at six months was used as the dependent variable.

To conduct the mediation analysis, we performed the Sobel test [34] to evaluate the non-zero indirect effect of the adherence to ≥ 4 dietary guideline recommendations as a mediator in relation to the objective clinical outcomes reported in the TEXT ME main results paper, including LDL-cholesterol, systolic BP and BMI; and intervention (TEXT ME intervention vs. standard care). All statistical tests were 2-tailed and a 5% significance level was used in all the analyses.

## Results

**Fig. 1:**
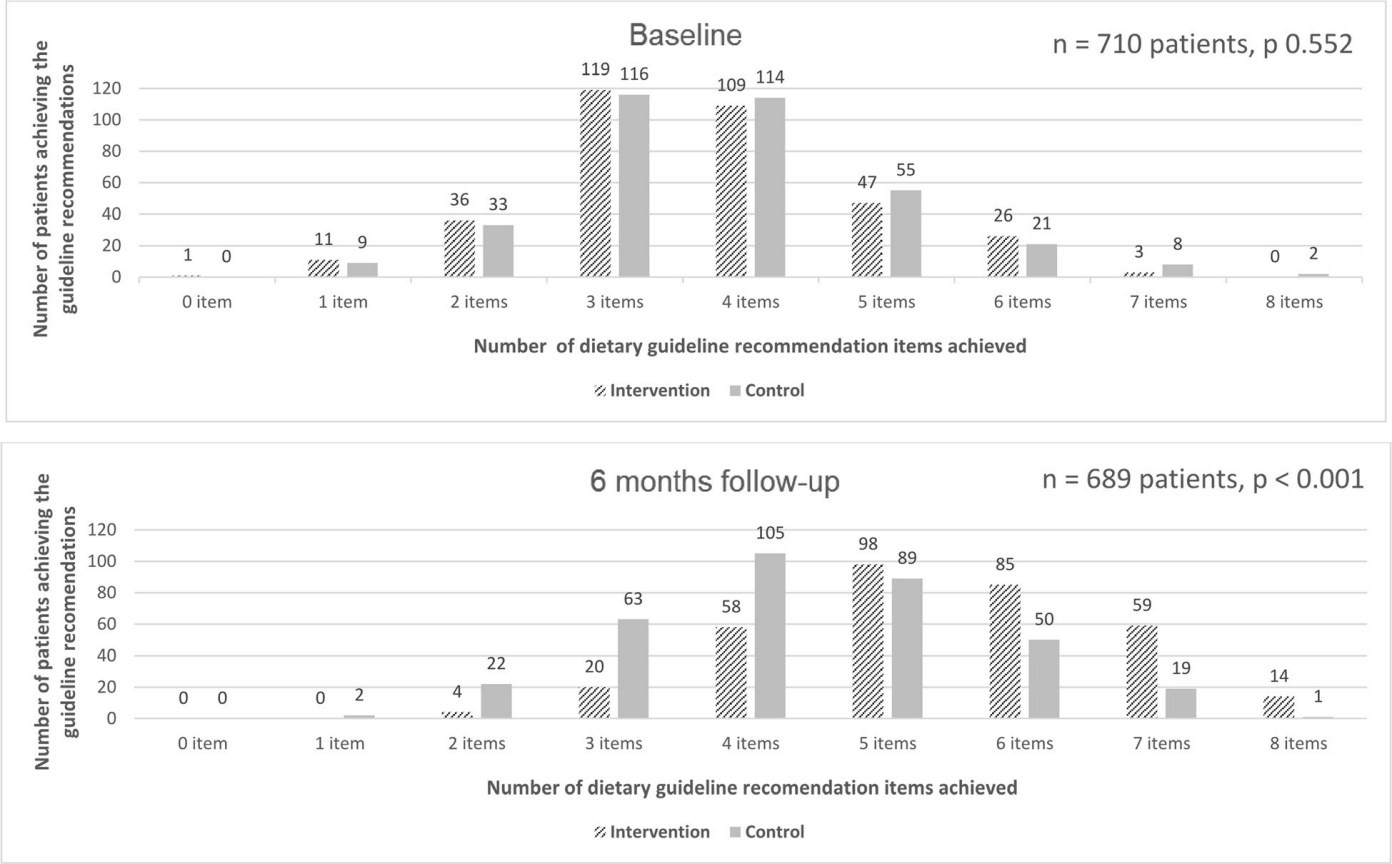
Distribution of patients in intervention and control groups by number of dietary guideline recommendation items achieved at baseline and six months

In the TEXT ME study, 710 patients were enrolled and randomised between September 2011 and November 2013. Dietary data were available for 710 patients at baseline. At six months, 21 patients did not have dietary data collected for several reasons (including unable-to-contact patients and those who died during the study period), hence the results of these diet analyses are presented using the dietary data available for 689 patients at six months. The baseline characteristics of patients per intervention group were previously reported. [28] At baseline, about half of the patients (*n* = 385/710, 54%) reported adhering to ≥ 4 key dietary guideline recommendations, and there was no significant difference between groups (intervention *n* = 185/352, 53% vs control *n* = 200/358, 56%, *p =* 0.376). In terms of patients’ characteristics according to the adherence to the dietary guideline recommendations at baseline, those adhering to < 4 recommendations had similar risk factor profiles and participation in cardiac rehabilitation compared with those adhering to ≥ 4 recommendations. However, those adhering to < 4 recommendations had lower level of education, higher proportion of smokers and higher mean BMI (Table 3). Only two patients were adhering to all eight key dietary guideline recommendations at baseline, both in the control group (Fig. 1). The most common dietary recommendations already achieved at baseline were use of unsaturated fat when cooking, consumption of ≤ 14 standard alcohol drinks per week, and use of margarine and unsaturated fat on bread, with 683 (96%), 653 (92%) and 534 (75%) patients meeting those recommendations, respectively. In contrast, only 19 (3%), 111 (16%), 113 (16%) reported consuming the recommended amounts for vegetables, fruit and fish, respectively. The mean number of dietary guideline recommendations achieved at baseline was 3.66 (95% confidence interval (CI) 3.53–3.79) and 3.78 (95% CI 3.65–3.91) in the intervention and control groups, respectively, and the difference was not statistically significant (*p* = 0.203).

**Table 3:** Patients demographics and risk factors stratified by adherence to dietary guideline recommendation items at baseline

	Adherence to ≥ 4 recommendation items (*n* = 385)	Adherence to < 4 recommendation items (*n* = 325)	*p value*	Overall cohort (*n* = 710)
Age, years, mean, (SD)	57.9 (8.77)	57.2 (9.64)	*0.268*	57.6 (9.18)
Males, *n* (%)	306 (79)	276 (85)	*0.060*	582 (82)
Education, years, mean (SD)	11.8 (3.64)	10.8 (3.19)	*< 0.001*	11.4 (3.48)
Ethnicity, *n* (%)			*0.100*	
European	241 (63)	232 (71)		473 (67)
South Asian	49 (13)	27 (8)		76 (11)
Other Asian	44 (11)	28 (9)		72 (10)
Middle Eastern	39 (10)	32 (10)		71 (10)
Other	12 (3)	6 (2)		18 (3)
Hypertension, *n* (%)	242 (63)	198 (61)	*0.597*	440 (62)
Diabetes, *n* (%)	124 (32)	105 (32)	*0.977*	229 (32)
Depression, *n* (%)	57 (15)	52 (16)	*0.660*	109 (15)
Current smoker, *n* (%)	174 (45)	203 (62)	*< 0.001*	377 (53)
BMI, mean (SD)	29.2 (5.52)	30.1 (6.37)	*0.044*	29.7 (5.93)
Cardiac rehabilitation attendance, *n* (%)	162 (43)	132 (42)	*0.683*	294 (43)

*BMI* body mass index, *SD* standard deviation

### Adherence to ≥ 4 dietary guideline recommendations at six months

Compared to baseline, both study groups increased the number of patients that reported adhering to ≥ 4 key dietary guideline recommendations at six months (Table 4). However, there was a bigger increase in the TEXT ME intervention group compared to the control group, with 93% (314/338) and 75% of patients (264/351) adhering to ≥ 4 recommendations, respectively (RR 1.23, 95% CI 1.15–1.31, *p* < 0.001). Fifteen patients reported adhering to all eight key dietary guideline recommendations at six months, being 14 of those in the intervention group and only one in the control group (Fig. 1). The mean number of dietary guideline recommendations achieved also increased in both groups, but there was a higher increase in the intervention group (mean number of recommendations achieved 5.40, 95% CI 5.26–5.54) compared to the control group (4.39, 95% CI 4.25–4.53), with a mean difference of 1.01 (95% CI 0.81–1.20, *p* < 0.001).

**Table 4:** Individual dietary guideline recommendation items at six months by study groups

	Text-message intervention group (*n* = 338)	Standard care group (*n* = 351)		
Guideline recommendation levels	*n* (%)	*n* (%)	RR (95% CI)	*p value*
1. Serves of vegetables per week				
≥ 35 serves per week	38 (11)	10 (3)	3.95 (2.00–7.79)	*< 0.001*
25–34 serves per week	49 (15)	21 (6)	2.42 (1.49–3.95)	*< 0.001*
15–24 serves per week	132 (39)	99 (28)	1.38 (1.12–1.71)	*0.003*
< 15 serves per week	119 (35)	221 (63)	0.56 (0.47–0.66)	*< 0.001*
2. Serves of fruits per week				
≥ 14 serves per week	165 (49)	85 (24)	2.02 (1.63–2.50)	*< 0.001*
10–13 serves per week	35 (10)	19 (5)	1.91 (1.12, 3.28)	*0.015*
6–9 serves per week	65 (19)	110 (31)	0.61 (0.47, 0.80)	*< 0.001*
< 6 serves per week	73 (22)	137 (39)	0.55 (0.43, 0.70)	*< 0.001*
3. Use of poly and monounsaturated fats^a^	334 (99)	345 (98)	1.01 (0.99–1.02)	*0.511*
4. Use of margarine and unsaturated fats on bread^a^	298 (88)	283 (81)	1.06 (1.00–1.12)	*0.056*
5. ≥ 300 g of fish per week^a^	152 (45)	91 (26)	1.83 (1.52–2.21)	*< 0.001*
6. ≤ 1 takeaway meals per week^b^	236 (70)	194 (55)	1.21 (1.09–1.34)	*< 0.001*
7. Salt intake control^a^	282 (83)	211 (60)	1.39 (1.26–1.52)	*< 0.001*
8. ≤ 14 standard alcoholic drinks per week^b^	320 (95)	322 (92)	1.02 (1.00–1.04)	*0.118*
Mean levels of consumption	Mean (95% CI)	Mean (95% CI)	Mean difference (95% CI)	*p value*
Serves of vegetables per week	19 (18–20)	13 (12–14)	5.94 (4.61–7.26)	< 0.001
Serves of fruits per week	12 (11–12.5)	8 (7–9)	3.80 (2.78–4.83)	< 0.001
Grams of fish per week^c^	228 (207–250)	159 (139–179)	69.70 (40.68–98.72)	< 0.001
Takeaway meals per week^c^	1.4 (1.2–1.6)	2.2 (1.9–2.5)	−0.87 (− 1.22 – − 0.51)	< 0.001
Standard alcoholic drinks per week^c^	2.4 (1.7–3.1)	3.1 (2.4–3.9)	−0.74 (− 1.75–0.26)	0.066

*CI* confidence interval, *RR* relative risk

^a^Randomised groups (intervention/control) have been compared using the log-binomial regression adjusted for corresponding baseline values as binary variables

^b^Randomised groups (intervention/control) have been compared using the log-binomial regression adjusted for corresponding baseline values as continuous variables

^c^Randomised groups (intervention/control) have been compared using the analysis of covariance adjusted for the corresponding baseline values

### Individual key dietary guideline recommendations at six months

At six months, patients in the TEXT ME intervention group reported consuming higher amounts of vegetables, fruits, and fish per week; less takeaway foods per week; as well as, controlling more their salt intake (Table 4), and therefore were adhering to the dietary guideline recommendations more often, when compared to the control group. There was a mean increase of 4.6 serves of vegetables per week, 4.9 serves of fruits per week and 121.7 g of fish per week in the intervention group at six months, while the control group had no increase in serves of vegetables and a mean increase of 1.0 serve of fruits and 30.8 g of fish. There was a trend towards a higher number of patients in the intervention group adhering to the guideline recommendations at six months for use of margarine and unsaturated fat on bread and consumption of ≤ 14 standard alcohol drinks per week when compared to control patients (Table 4); however, this difference was not statistically significant.

### TEXT ME intervention effect by sub-groups

In the sub-group analysis, there were no significant differences on adherence to ≥ 4 dietary guideline recommendations across the subgroups tested (by age, sex, education, BMI, smoking and cardiac rehabilitation attendance), suggesting that the TEXT ME intervention had a similar effect in all sub-groups (Table 5).

**Table 5:** Sub-group analysis of the impact of the TEXT ME intervention on adherence to ≥ 4 dietary guideline recommendation items at six months

	N (Intervention / Standard care)	Text-message intervention	Standard care	RR (95% CI)	*p* value interaction
Age					
> 60 years	147/144	139 (94.6%)	114 (79.2%)	1.19 (1.09–1.31)	0.400
≤ 60 years	191/207	175 (91.6%)	150 (72.5%)	1.26 (1.15–1.39)	
Sex					
Female	62/60	57 (91.9%)	44 (73.3%)	1.23 (1.15–1.32)	0.850
Male	276/291	257 (93.1%)	220 (75.6%)	1.25 (1.06–1.49)	
Education^a^					
> 13 years	59/81	57 (96.6%)	60 (74.1%)	1.30 (1.14–1.50)	0.378
≤ 13 years	278/267	256 (92.1%)	202 (75.7%)	1.22 (1.13–1.31)	
BMI					
≥ 25 kg/m^2^	264/278	246 (93.2%)	206 (74.1%)	1.26 (1.16–1.36)	0.305
< 25 kg/m^2^	74/73	68 (91.9%)	58 (79.5%)	1.16 (1.01–1.32)	
Smoking					
Yes	177/190	157 (88.7%)	138 (72.6%)	1.16 (1.06–1.26)	0.089
No	161/161	157 (97.5%)	126 (78.3%)	1.29 (1.17–1.43)	
Cardiac rehabilitation attendance^b^					
Yes	151/143	144 (95.4%)	118 (82.5%)	1.22 (1.10–1.35)	0.767
No	186/208	169 (90.9%)	146 (70.2%)	1.25 (1.14–1.36)	

BMI, body mass index; CI, confidence interval; RR, relative risk

^a^4 patients with missing data on education

^b^1 patient with missing data on cardiac rehabilitation attendance

### Mediation analysis

In the mediation analysis, there were significant mediational effects of the increase in adherence to dietary guideline recommendations for the association between the TEXT ME intervention and the levels of LDL-cholesterol (*t* = 3.39, *p* < 0.001) and BMI (*t* = 2.83, *p* = 0.005) at six months follow-up, where the increase in adherence to dietary guideline recommendations mediated the association between the intervention and LDL-cholesterol and BMI by 68% and 47%, respectively. However, there was no significant mediational effect on systolic BP (*t* = 0.004, *p* = 0.996).

## Discussion

In this analysis, we present the results of the impact of a simple lifestyle-focused text-messaging intervention on adherence to heart-healthy dietary guideline recommendations, in a high-risk population of patients with CHD. The results herein presented are a pre-specified secondary outcome of the TEXT ME study. At six months, 93% of patients in the TEXT ME intervention group reported they were adhering to at least half of the eight dietary guideline recommendations compared to 75% of patients in the control group. Specifically, consumption of vegetables, fruits, and fish was higher in the intervention patients compared to control patients, whereas takeaway foods and salt were consumed less. The effects of the text-messaging intervention were consistent by age, gender, education level, baseline BMI, smoking status and participation in cardiac rehabilitation. In addition, effects of the text-messaging intervention on LDL-cholesterol and BMI were partially mediated by improvements in adherence to the dietary guideline recommendations, but not the effects on systolic BP.

It is important to highlight that although the text-messaging intervention improved adherence to the dietary guideline recommendations, the proportions of patients meeting the recommendations for consumption of vegetables, fruits and fish were still low, with less than 15% of patients achieving the recommendations for vegetables and less than 50% for fruits and fish at six-months follow-up. These results are similar to other studies showing low consumption of vegetables and fruits in people with CHD, [14, 35] and are also comparable to the results of the Australian National Health Survey 2014–15, which showed that only 7.0 and 49.8% of the population are meeting the recommended daily consumption of vegetables and fruits, respectively. [36] In contrast, the proportion of patients that reported using unsaturated fat oils for cooking was already very high at baseline, similar to proportions shown in another study, [14] and hence unsurprisingly, there was no further improvement with respect to this variable. Similarly, though there were no text-messages specifically addressing alcohol consumption, the proportion of patients consuming less than two standard alcoholic drinks per week was very high at baseline and did not differ between groups at six-months follow-up. At baseline, patients adhering to < 4 of the eight dietary guideline recommendations assessed in this study had lower education, higher BMI and higher proportion of smokers. Other studies have also reported associations between having a poorer diet with lower education [4, 37–39] and other unhealthy behaviours, [40] such as being a smoker [3, 6, 14, 37, 38, 41] and having a higher BMI. [3, 6, 37, 38] Importantly, in our analysis, there were improvements in adherence to the dietary guideline recommendations in sub-groups with both lower and higher education, lower and higher BMI, and smokers and non-smokers with no heterogeneity identified.

Only a few studies have previously assessed text-messaging to improve diet in people with CHD. In a prior study of a similar intervention, Pfaeffli and colleagues conducted a RCT on the impact of mobile technology intervention that included text-messages in 123 patients with CHD. In this study, the authors found that the intervention was associated with improvements in the consumption of vegetables and fruits when compared to control at three and six months; however, there were no significant differences in alcohol intake. [42] Other studies that have investigated interventions that included text-messages alone or text-messages plus emails or phone counselling have shown mixed results. [43–50] However, these were small studies conducted in primary prevention populations, including young adults and overweight and obese people, with a primary focus on weight management or CVD primary prevention. In contrast, the TEXT ME study had a secondary prevention population, and therefore, given their previous history of CHD, the patients in the TEXT ME study might have been more motivated to lifestyle changes when compared to these other primary prevention populations.

Importantly, the improvements in adherence to the dietary guideline recommendations demonstrated in this analysis support and are related to the main findings of the TEXT ME study [28]. These improvements were shown to have partially mediated the significant reductions in LDL-cholesterol and BMI found in the intervention group compared to the control group. The TEXT ME study has a unique strength as our text-messaging intervention was designed to target multiple lifestyle behaviours, different from other studies that focussed on individual risk factors such as smoking [51] or overweight. [52] It is also of note that the process evaluation that accompanied the TEXT ME study identified that the diet messages were most valued. [53] Coupled with the low cost of delivery, [54] these findings argue further for such text-messaging programmes having excellent potential as scalable solutions to be delivered to many people with CHD.

This study has several limitations. First, the assessment of the dietary data relied on self-reported information and was potentially subject to bias due to social desirability responding, which is a known limitation of self-report research. [55] However, self-reported measures of dietary intake are the most commonly used methods [56]. Second, although we used a questionnaire very similar to the WHO STEPS instrument, which is a simple, standardised method of collecting dietary data across countries, [57] such short dietary assessment instruments do not provide a comprehensive assessment of diet and are also prone to systematic error. The study diet questionnaire only assessed eight of the dietary guideline recommendations and did not cover all aspects of diet, such as consumption of grains, milk and dairy products, and discretionary food choices. However, the dietary guideline recommendations in Australia are not specific to a population with CVD, therefore, we focussed on assessing food items that have been associated with lower CVD. Third, the assessment of consumption of takeaway foods did not provide details of the type of takeaway food consumed, e.g. high vs. low saturated fat. Nevertheless, it is known that eating away from home is associated with poor-quality diet. [58, 59] Fourth, the choice of assessing the proportions of patients adhering to ≥ 4 dietary guideline recommendations was somewhat arbitrary and only full adherence to the dietary guideline items was considered; however, we also reported means and distributions. Finally, this study had a short follow-up of six months and it was conducted in one centre in Australia, hence, our results might not be sustained for a longer period and be generalisable to other contexts.

## Conclusion

A lifestyle-focused text-message programme improved the proportion of patients who reported adhering to the dietary guideline recommendations, as well as, improved individually the consumption of vegetables, fruits, fish, takeaway foods and salt intake when compared to standard care at six months in a population of patients with CHD; however, it had no effects on unsaturated fats and alcohol consumption. Importantly, the improvements in adherence to dietary guideline recommendations partially mediated improvements in LDL-cholesterol and BMI, but not the observed reduction in systolic BP. This text-messaging programme is a simple and scalable intervention that could be used as a strategy to improve diet in people with CHD as a stand-alone intervention or in combination with other programmes, such as cardiac rehabilitation. This promising intervention may also be transferrable to other patient populations, such as those with other chronic diseases.

## Additional files

Additional file 1: TEXT ME diet questionnaire. (PDF 432 kb)

Additional file 2: WHO STEPS diet instrument. (PDF 294 kb)
