# Supplementary material for: The effects of a lifestyle-focused text-messaging intervention on adherence to dietary guideline recommendations in patients with coronary heart disease: an analysis of the TEXT ME study
Source: Int J Behav Nutr Phys Act. 2018 May 23;15:45. doi: 10.1186/s12966-018-0677-1 (PMC5967045; doi:10.1186/s12966-018-0677-1)
Supplement: Supplementary file 1 — TEXT ME diet questionnaire. (PDF 432 kb) [file 12966_2018_677_MOESM1_ESM.pdf]

TEXTME

Form 1.6 -Diet

Visit 1 - 0 weeks

Centre number Patient number

StudyID |__|__|__|-|__|__|__|__|

Participant Initials |__| |__|__|__|__|

- Answer every main question. Answer every supplementary question *(If Yes* or *If No)* as indicated by arrows.
- Tick boxes. Enter all dates as dd/mm/yyyy
- Enter * if data will never be available. Enter 77 if participant responds 'don't know'

1. Fruit and vegetable consumption

1.01 |__|__| In a typical week, on how many days do you eat fruit? (Number of days)

1.02 |__|__| How many servings of fruit do you eat on one of those days? (Number of servings)

1.03 |__|__| In a typical week, on how many days do you eat vegetables? (Number of days)

1.04 |__|__| How many servings of vegetables do you eat on one of those days? (Number of servings)

2. Fish consumption

2.01 |__|__|__|__| In a typical week how many grams of fish do you eat in a typical week? (1 serving is about 150g)

3. Oil and fat consumption

3.01 What type of oil or fat is most often used for cooking in your household?

- ☐ Vegetable oil
- ☐ Lard or suet
- ☐ Butter
- ☐ Margarine
- ☐ Ghee
- ☐ Olive Oil
- ☐ Other, please specify ____________________

3.02 What type of oil or spread is most often used on your bread/ toast?

- ☐ None
- ☐ Vegetable
- ☐ Margarine
- ☐ Cholesterol-lowering spread, e.g. Proactiv$_{TM}$
- ☐ Other, please specify ____________________

3.03 |__|__| How many slices of 'buttered' bread or toast do you eat per week?

4. Place of preparation

4.01 |__|__| On average, how many meals per week do you eat that were not prepared at a home?

By meals I mean breakfast, lunch and dinner

5. Salt intake behaviours

	yes	no			
5.01	Y	N	Do you do anything on a regular basis to control your salt/sodium intake?		
5.02	→		Y	N	Avoid/ minimize processed foods
5.03			Y	N	Look at the salt/ sodium labels on foods
5.04			Y	N	Do not add salt to food at the table
5.05			Y	N	Buy low salt alternatives
5.06			Y	N	Buy low sodium alternatives
5.07			Y	N	Do not add salt when cooking
5.08			Y	N	Use spices other than salt when cooking
5.09			Y	N	Avoid eating out
5.10			Y	N	Other, please specify ____________________
